# Distribution of raphespinal fibers in the mouse spinal cord

**DOI:** 10.1186/s12990-015-0046-x

**Published:** 2015-07-16

**Authors:** Huazheng Liang, Shaoshi Wang, Richard Francis, Renee Whan, Charles Watson, George Paxinos

**Affiliations:** Neuroscience Research Australia, 139 Barker Street, Randwick, NSW 2031 Australia; Department of Neurology, Branch of Shanghai First People’s Hospital, Shanghai, 200081 China; Biomedical Imaging Facility, The University of New South Wales, Sydney, NSW 2052 Australia; Health Sciences Dean Research, Faculty of Health Sciences, Curtin University, Shenton Park Campus, Perth, WA 6102 Australia; School of Medical Sciences, The University of New South Wales, Sydney, NSW 2052 Australia

**Keywords:** Hindbrain, Raphe nuclei, Reticular nuclei, Serotonin, Spinal cord, Raphespinal tract, CLARITY, Anterograde tracing

## Abstract

**Background:**

Serotonergic raphespinal neurons and their fibers have been mapped in large mammals, but the non-serotonergic ones have not been studied, especially in the mouse. The present study aimed to investigate the termination pattern of fibers arising from the hindbrain raphe and reticular nuclei which also have serotonergic neurons by injecting the anterograde tracer BDA into them.

**Results:**

We found that raphespinal fibers terminate in both the dorsal and ventral horns in addition to lamina 10. There is a shift of the fibers in the ventral horn towards the dorsal and lateral part of the gray matter. Considerable variation in the termination pattern also exists between raphe nuclei with raphe magnus having more fibers terminating in the dorsal horn. Fibers from the adjacent gigantocellular reticular nucleus show similar termination pattern as those from the raphe nuclei with slight difference. Immunofluorescence staining showed that raphespinal fibers were heterogeneous and serotoninergic fibers were present in all laminae but mainly in laminae 1, 2, medial lamina 8, laminae 9 and 10. Surprisingly, immunofluorescence staining on clarified spinal cord tissue revealed that serotoninergic fibers formed bundles regularly in a short distance along the rostrocaudal axis in the medial part of the ventral horn and they extended towards the lateral motor neuron column area.

**Conclusion:**

Serotonergic and non-serotonergic fibers arising from the hindbrain raphe and reticular nuclei had similar termination pattern in the mouse spinal cord with subtle difference. The present study provides anatomical foundation for the multiple roles raphe and adjacent reticular nuclei play.

**Electronic supplementary material:**

The online version of this article (doi:10.1186/s12990-015-0046-x) contains supplementary material, which is available to authorized users.

## Background

Raphespinal neurons in the hindbrain are the major source of serotoninergic fibers to the spinal cord and they have been well known for the involvement of biphasic pain modulation [1–5]. Physiological and pharmacological studies have demonstrated the increased release of serotonin in the dorsal horn of the spinal cord after electrical stimulation of the raphe nuclei in the hindbrain [3, 6, 7]. Retrograde tracing studies also have shown that raphe nuclei in the hindbrain project to the entire spinal cord [8–12]. During the development, the raphe nuclei are among the early groups of neurons projecting to the spinal cord, which suggests their involvement in the development of locomotion at the early stage of life [13].

It has been reported that in the rat and opossum serotonergic raphespinal fibers terminate heavily not only in the dorsal horn [14–16] but also in the intermediate zone [15, 17, 18] and the ventral horn [15, 19], even lamina 10 [14, 15].

Apart from serotoninergic neurons, raphe nuclei also contain other phenotypes of neurons [20–22], such as GABAergic [23, 24] and peptidergic neurons [21]. These non-serotoninergic neurons are about 1/3 of the raphe magnus (RMg) neurons which project to the spinal cord [20]. Their fibers are present in all laminae of the rat spinal cord [15]. However, the distribution of fibers arising from these non-serotonergic neurons has not been studied in the mouse. In the retrograde study on the mouse, it has been shown that the alpha/ventral part of the gigantocellular reticular nucleus (GiA/V) and the lateral paragigantocellular reticular nucleus (LPGi) (these neurons are more likely to be in the lateral part of GiA/V) also contain serotonergic spinal cord projecting neurons [22]. We aimed to map the termination pattern of the non-serotonergic fibers arising from these raphe nuclei as well as those from the adjacent reticular nuclei in the mouse spinal cord by injecting biotinylated dextran amine (BDA) and double labelling these BDA labelled fibers with serotonin antibody. In comparison, the serotonergic fibers were demonstrated with immunofluorescence and CLARITY on the mouse spinal cord segments.

## Results

### BDA labeled fibers in the spinal cord

**Figure 1:**
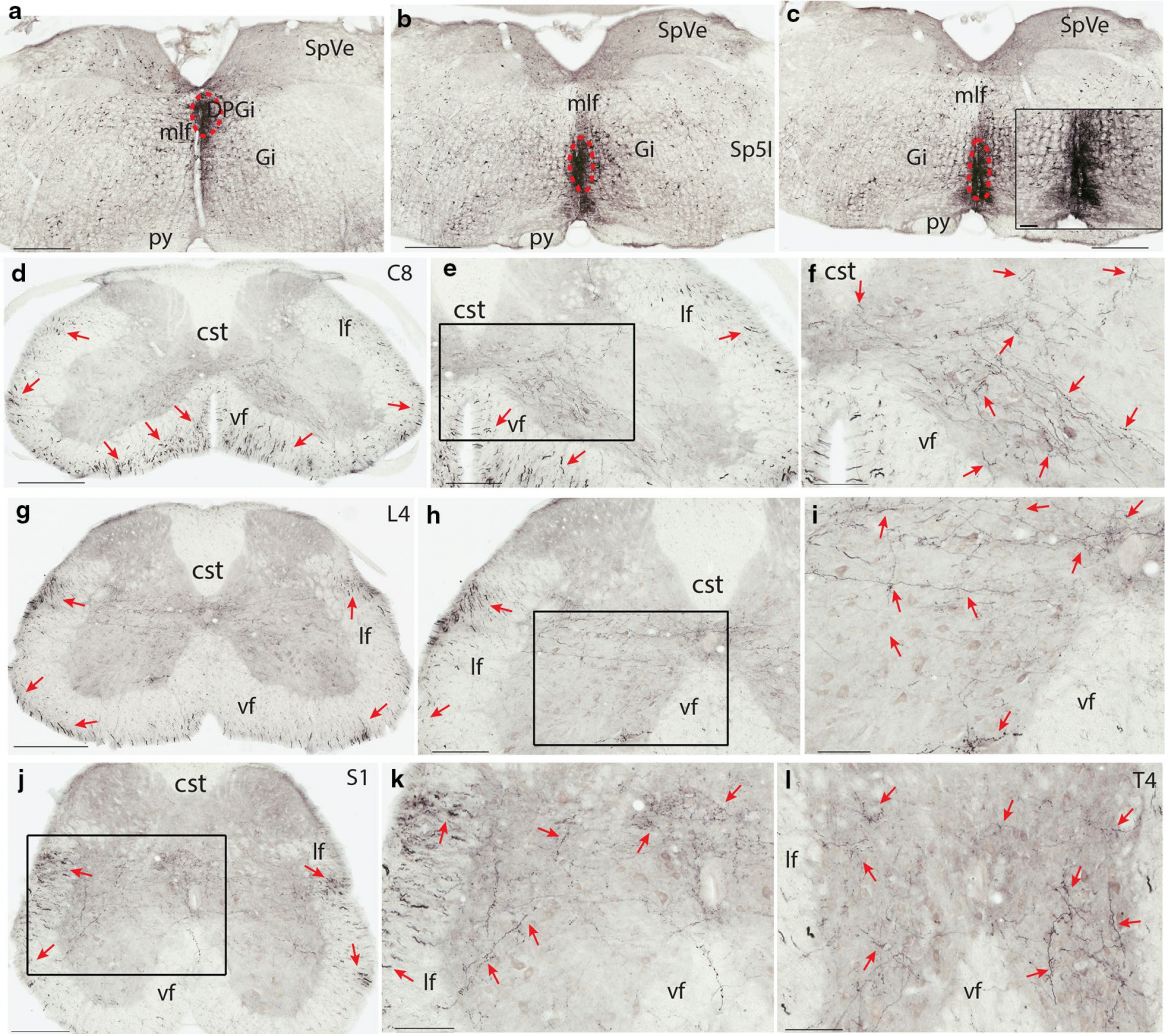
Termination pattern of BDA labeled fibers arising from RPa and ROb. **a**–**c** An injection site in RPa and ROb, which involved the dorsal paragigantocellular reticular nucleus (*red dashed circle*). **d** A C8 section showing the labelled fibers in both the ventral and lateral funiculi (*red arrows*). **e** A C8 section showing labeled fibers in both the ventral and lateral funiculi (*red arrows*) and the medial lamina 7, and 8, and lamina 10. **f** A higher magnification of the *rectangular area* in **e**. Note labeled fibers in medial laminae 7, 8, and 9, and 10. **g** An L4 section showing labeled fibers in both the ventral and lateral funiculi (*red arrows*). Note there are more fibers in the lateral funiculus than in the ventral funiculus. **h** An L4 section showing labeled fibers in the lateral funiculus and in laminae 6–10. **i** A higher magnification of the *rectangular area* in **h**. **j** An S1 section showing labeled fibers in the lateral and ventral funiculi and in laminae 6–10. Note there are more fibers in the lateral funiculus than in the ventral funiculus. **k** A higher magnification of the *rectangular area* in **j**. **l** A T4 section showing labeled fibers in laminae 7–10 (*red arrows*). The *scale bar* 500 μm in **a**–**c**, 400 μm in **d** and **g**, 200 μm in **e**, **h**, and **j**, 100 μm in **f**, **i**, **k**, **l** and the microphotograph in **c**.

After injecting BDA to the raphe pallidus nucleus (RPa) and raphe obscurus nucleus (Rob) nuclei at the level of the rostral vagus nerve nucleus (10N) (Figure 1a–c), labeled fibers were found in the ventral portion of the ventral funiculus, and to a lesser extent, in the lateral funiculus bilaterally in the cervical segments (Figure 1d–f). In the thoracic and lower segments, the density of labeled fibers in the ventral funiculus decreased (82 ± 9 in cervical, 43 ± 7 in thoracic, 40 ± 6 in lumbar, 15 ± 3 in sacral cord), whereas the density of labeled fibers in the dorsolateral funiculus increased (19 ± 4 in cervical, 33 ± 6 in thoracic, 35 ± 5 in lumbar, 41 ± 5 in sacral) (Figure 1g–l).

In the gray matter, labeled fibers and boutons were constantly found in the medial part of laminae 7–10 (Figure 1d–l). These fibers and boutons were more laterally distributed in more caudal segments and density of them tapered caudally. In the ventral and lateral motor columns, boutons were also constantly observed though the density of labeled fibers was low. In the cervical cord, a small number of fibers extended from the medial lamina 7 to the lateral part of lamina 6, and even laminae 4 and 5 (Figure 1f). In the thoracic and lower segments, there were more fibers and boutons in these regions and they were continuous with those densely labeled fibers and boutons surrounding the central canal (Figure 1g–k). In the thoracic cord, some terminals were also observed in the intermediolateral column though the density of the labeled fibers and boutons was low (Figure 1l).

**Figure 2:**
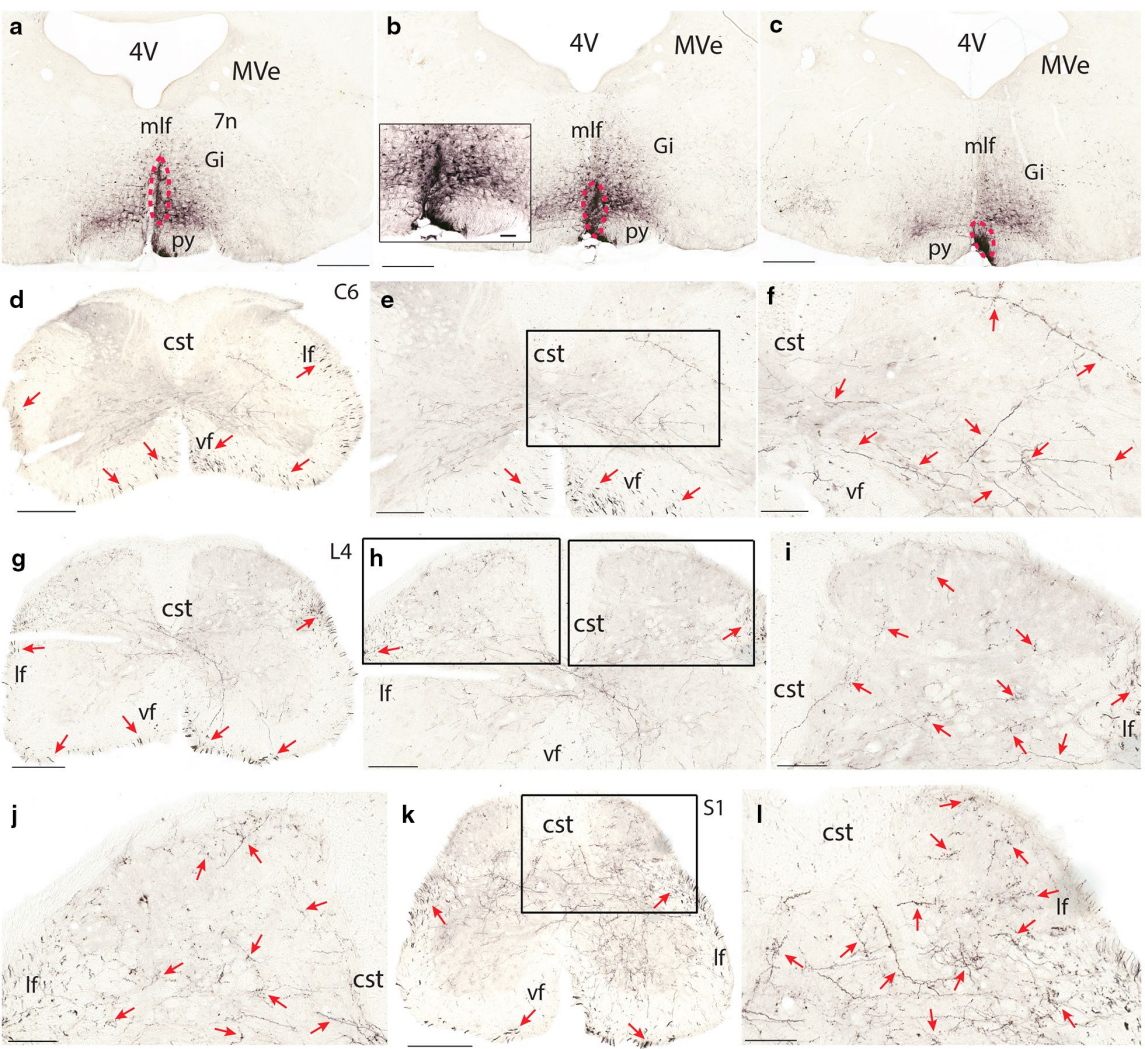
Termination pattern of BDA labeled fibers arising from RMg. **a**–**c** an injection site in RMg (*red dashed circle*). **d** A C6 section showing the labeled fibers in both the ventral and lateral funiculi (*red arrows*). **e** A C6 section showing labeled fibers in the lateral funiculi (*red arrows*) and lamina 7, 8, 9, and 10. **f** A higher magnification of the *rectangular area* in **e**. Note labeled fibers in laminae 7–10. **g** An L4 section showing labeled fibers in both the ventral and lateral funiculi (*red arrows*). **h** An L4 section showing labeled fibers in bilateral dorsal horn and the dorsal part of the ventral horn. **i** A higher magnification of the *right rectangular area* in **h**. Note labeled fibers in laminae 1, 2, 4, and 5. **j** A higher magnification of the *left rectangular area* in **h**. Note few labeled fibers in lamina 3. **k** An S1 section showing labeled fibers in the lateral and ventral funiculi and in all laminae. Note there are more fibers in the lateral funiculus than in the ventral funiculus. **l** A higher magnification of the *rectangular area* in **k**. The *scale bar* 500 μm in **a**–**c**, 400 μm in **d**, 300 μm in **g** and **k**, 200 μm in **e**, and **h**, 100 μm in **f**, **i**, **j**, **l**, and the microphotograph in **b**.

BDA injections into the rostral raphe nucleus- RMg (Figure 2a–c) led to similar results as those from RPa and ROb injections. However, there were much more fibers and boutons in laminae 1–5 in the lumbar and sacral segments though there were few fibers and boutons in lamina 3 (Figure 2g–l). In the lumbar and sacral segments, there were also more fibers and boutons in the medial part of laminae 7–10 (7 ± 2 in cervical, 9 ± 2 in thoracic, 13 ± 2 in lumbar, and 14 ± 3 in sacral cord in a 100 μm × 100 μm area) (Figure 2g–l).

**Figure 3:**
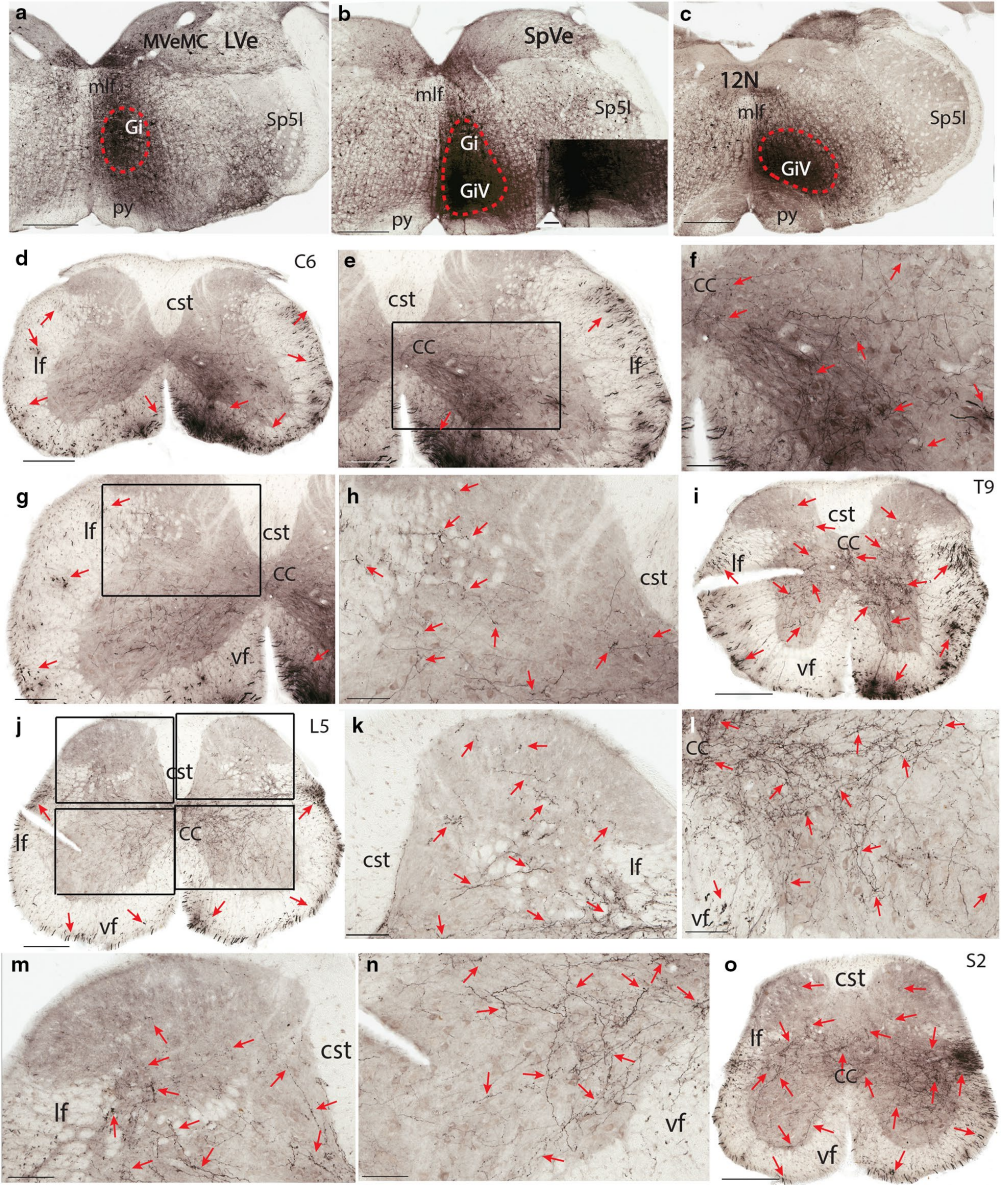
Termination pattern of BDA labeled fibers arising from GiV. **a**–**c** An injection site in GiV (*red dashed circle*) which involved the ventral part of the Gi. **d** A C6 section showing the labeled fibers in both the ventral and lateral funiculi (*red arrows*). Note the ipsilateral predominance, especially in the ventral funiculus (the *right* is the ipsilateral side and the *left* is the contralateral side). **e** A C6 section showing labeled fibers in both the ventral and lateral funiculi (*red arrows*) and in lamina 6–10 on the ipsilateral side. **f** A higher magnification of the *rectangular area* in **e**. Note densely labeled fibers in medial laminae 7 and 8, and 9, and 10. **g** A C6 section showing labeled fibers in both the ventral and lateral funiculi (*red arrows*) and lamina 6–10 on the contralateral side. **h** A higher magnification of the *rectangular area* in **g**. Note labeled fibers in medial laminae 5–7 and 10. **i** A T9 section showing labeled fibers in both the lateral and ventral funiculi and in laminae 5, 7–10 (*red arrows*). Note the density of labeled fibers in the ventral funiculus is higher than that of the lateral funiculus. **j** An L5 section showing labeled fibers in both the ventral and lateral funiculi (*red arrows*). Note the similar density of labeled fibers in the ventral and lateral funiculi. **k** An L5 section showing labeled fibers in the ipsilateral dorsal horn (the *top right rectangular area* in **j**). Note labeled fibers in laminae 3–6, and to a lesser extent in laminae 1–3. **l** An L5 section showing labeled fibers in the ipsilateral ventral horn (the *lower right rectangular area* in **j**). Note more labeled fibers in laminae 8 and 9 than those from raphe nuclei. **m** An L5 section showing labeled fibers in the contralateral dorsal horn (the *top left rectangular area* in **j**). Note labeled fibers in laminae 4–6, and to a lesser extent in lamina 3. **n** An L5 section showing labeled fibers in the contralateral ventral horn (the *lower left rectangular area* in **j**). Note labeled fibers in laminae 7–9. **o** An S2 section showing labeled fibers in the lateral and ventral funiculi and in all laminae except in the ipsilateral laminae 1–3. Note the density of labeled fibers in the lateral funiculus is higher than that in the ventral funiculus. The *scale bar* 500 μm in **a**–**c**, 400 μm in **d**, 300 μm in **i**, **j**, and **o**, 200 μm in **e**, **g**, **k**, and **m**, 100 μm in **f**, **h**, **l**, **n**, and the microphotograph in **b**.

In contrast to raphe nuclei injections, BDA injections to the adjacent GiV led to more labeled fibers on the ipsilateral side (Figure 3a–c). In the white matter of the cervical cord, there were more fibers in the ventral funiculus than in the lateral funiculus (Figure 3d). The density of labeled fibers dropped in lower segments and there were more of them in the dorsolateral funiculus than in the ventral funiculus in the lumbar and sacral cord (Figure 3j, o). In the gray matter, BDA labeled fibers and boutons were similar to those arising from RPa and Rob. However, there were more fibers and boutons extending from the medial laminae 7 and 8 towards the lateral laminae 6 and 7 (Figure 3d–o). In addition, there was a larger number of fibers and boutons in the medial laminae 7–10 compared to that of fibers and boutons arising from RPa and Rob (Figure 3d–g, j–m, o). Furthermore, there was a small band of fibers extending from the lateral lamina 6 towards the medial part of laminae 4 though the density of their boutons was low (Figure 3j, k, o). In laminae 1–3, labeled fibers were hardly found. Double labeling with serotonin antibody showed that there was no overlap between Alexa fluor 488 labeled serotonin fibers and Alexa fluor 594 labeled raphespinal fibers (data not shown).

**Figure 4:**
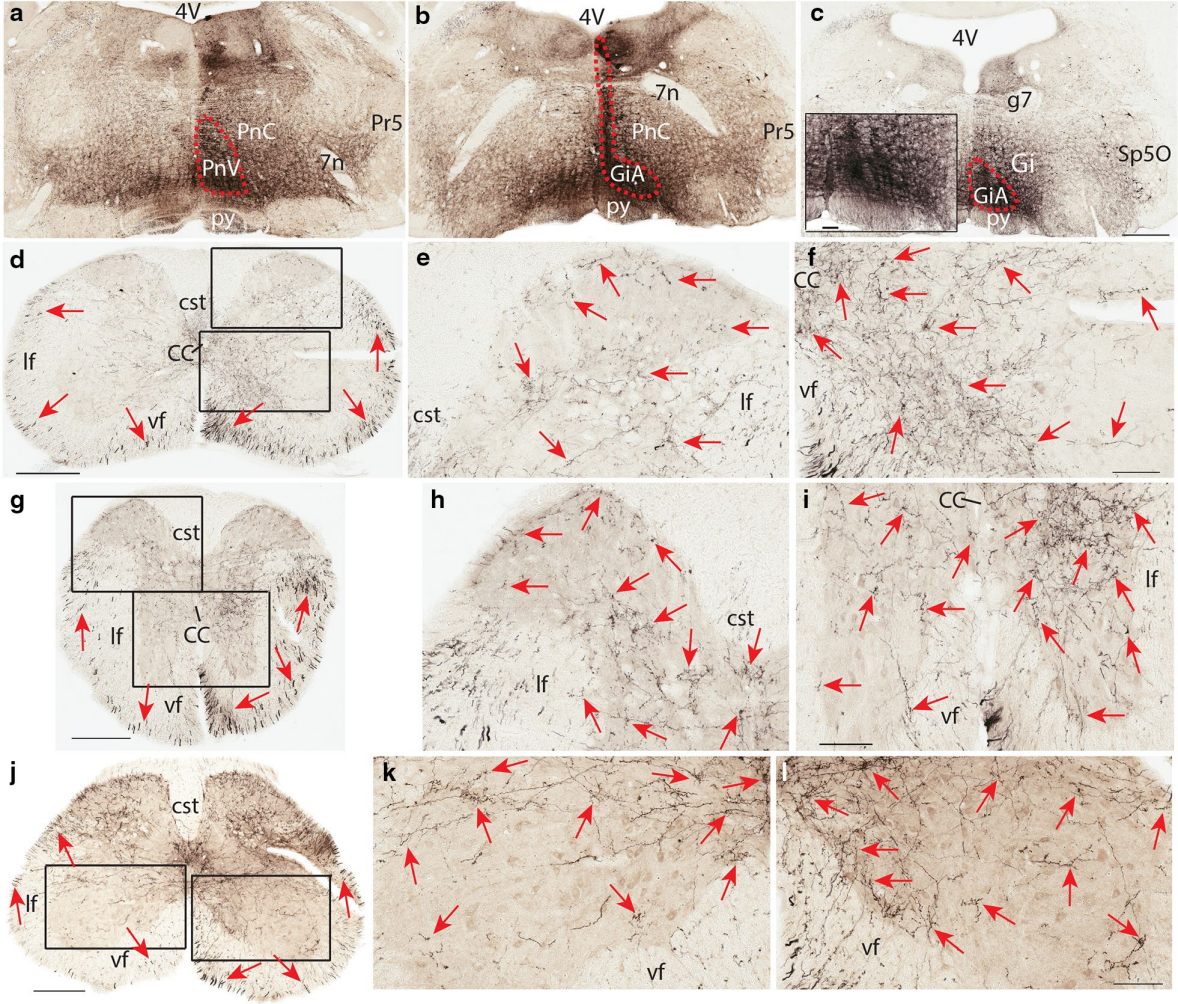
Termination pattern of BDA labeled fibers arising from the rostral part of GiA. **a**–**c** An injection site in GiA, which involved the medial portion of the caudal part of the pontine reticular nucleus (*red dashed circle*). **d** A C5 section showing the labelled fibers in both the ventral and lateral funiculi (*red arrows*) on both sides with an ipsilateral predominance (the *right* is the ipsilateral side and the *left* is the contralateral side). **e** A higher magnification of the dorsal *rectangular area* in **d** showing labeled fibers in laminae 1–6 with a low density of fibers in lamina 3 on the ipsilateral side (*red arrows*). **f** A higher magnification of the ventral *rectangular area* in **d** showing densely labeled fibers in laminae 8, 10, and the medial part of laminae 7, 9 on the ipsilateral side (*red arrows*). **g** A T5 section showing labeled fibers in both the ventral and lateral funiculi (*red arrows*). Note the density of labeled fibers in the dorsolateral funiculus is higher than that in the cervical cord. **h** A higher magnification of the dorsal *rectangular area* in **g** showing labeled fibers in laminae 1–7, and 10 with a low density of fibers in lamina 3 on the contralateral side (*red arrows*). **i** A higher magnification of the ventral *rectangular area* in **g** showing densely labeled fibers in laminae 7–10 with an ipsilateral predominance (*red arrows*). **j** An L5 section showing labeled fibers in the lateral and ventral funiculi and in all laminae of the gray matter. Note there are more fibers in the lateral funiculus than in the ventral funiculus. **k** A higher magnification of the *left rectangular area* in **j** showing labeled fibers in laminae 7–10 with more fibers in the lateral lamina 9 (*red arrows*). **l** A higher magnification of the *right rectangular area* in **j** showing densely labeled fibers in laminae 7, 8, 10, and the medial lamina 9 with few fibers in the lateral lamina 9 (*red arrows*). The *scale bar* 500 μm in **a**–**c**, 400 μm in **d** and **j**, 300 μm in **g**, 100 μm in **e**, **f**, **h**, **i**, **k**, **l**, and the microphotograph in **c**.

BDA injections to the rostral portion of the GiA resulted in similar fiber distribution in the spinal cord except that there were more BDA labeled fibers in the entire dorsal horn (Figure 4a–l).

**Figure 5:**
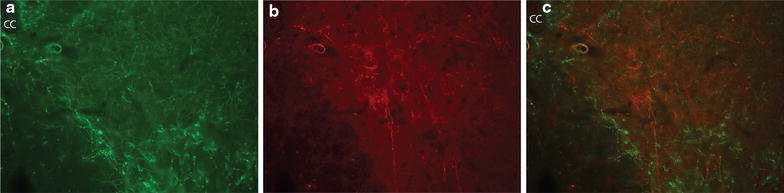
Double labeling of BDA labeled fibers with serotonin antibody. **a** A section showing serotoninergic fibers in the medial ventral horn of C5. **b** The same section in **a** showing BDA labeled fibers. **c** Overlap of **a** and **b**. The *scale bar* 200 μm.

Double labeling with serotonin antibody on BDA labeled fibers did not show any overlap (Figure 5a–c). This will be discussed later in the “Discussion”.

Control injections into the cerebellum labeled few fibers in the contralateral spinal cord. BDA injections into the cisterna magna did not reveal label fibers in the spinal cord (data not shown).

### Serotonergic fibers in the spinal cord

**Figure 6:**
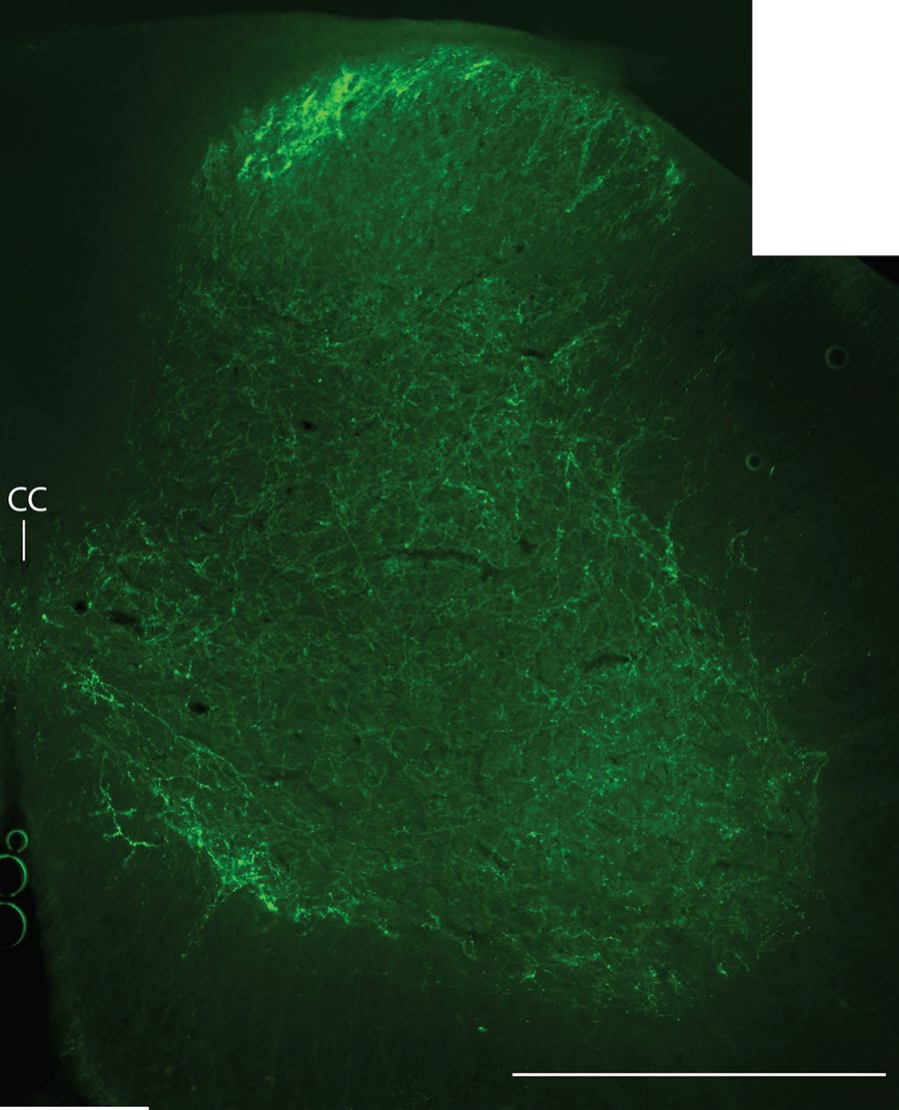
Immunofluorescence staining with serotonin antibody in a C5 section (a photomerged section under 10 times magnification). Note densely labeled fibers in laminae 1, 2, the medial lamina 8, and 9 (motor neuron column area), and 10 (the central canal area). All the other laminae also have sparsely labeled fibers. The *scale bar* 500 μm.

Immunofluorescence staining showed that serotoninergic fibers and boutons were mainly present in laminae 1, 2, 10, and the area surrounding the motor neuron columns. In other laminae of the spinal cord, labeled fibers and boutons were widely spread and the density of them was low (ranging from 35 ± 6 to 74 ± 9 axons/200 μm × 200 μm area) (Figure 6).

In the CLARITY (*C*lear, *L*ipid-exchanged, *A*crylamide-hybridized *R*igid, *I*maging/immunostaining compatible, *T*issue h*Y*drogel) spinal cord, serotonergic fibers entered the gray matter from both the lateral and ventral funiculi. Densely labeled fibers were also found in laminae 1, 2, 10, and in areas surrounding the motor neuron columns. Those fibers in the gray matter travelled along the rostrocaudal axis and terminated along their path (Additional file 1: Video S1). In the dorsal horn, the majority of the fibers were thin and only a small number of thick fibers were observed, especially in lamina 4 (Additional file 2: Video S2). In laminae 5–8, a small number of thick fibers were also found and they were mainly found in the lateral half of the gray matter. In lamina 9, densely packed fibers were found in the medial part of it. These fibers formed a bundle along the rostrocaudal axis of the spinal cord and issued the first branch at a regular distance, which gave off smaller branches and formed an axonal tree with its terminals in the motor neuron columns (Additional file 3: Video S3).

## Discussion

The present study showed that raphespinal fibers were widely distributed in the gray matter of the entire spinal cord. There was some variation between the fiber terminals arising from different parts of the raphe nuclei, but those in the medial laminae 7–10 were constantly present in all segments. Adjacent reticular nuclei had similar fiber distribution in the spinal cord with an ipsilateral predominance. Fiber terminals in medial laminae 7–10 and the fiber stripe in the lateral laminae 4–7 were always present in all segments. Serotonergic fibers were slightly different from these fibers revealed by BDA injections. They were mainly present in the superficial layers of the dorsal horn, the area surrounding the central canal and the motor neuron columns. In the ventral part of the spinal cord, the serotonergic fibers were forming big bundles regularly. They extended from the medial part of the lamina 9 towards the lateral motor neuron column and gave collaterals along the path.

### Fiber terminals arising from the raphe nuclei

The present study showed that raphespinal fibers originating from different raphe nuclei varied slightly in their distribution in the spinal cord. While RPa and ROb in the caudal hindbrain terminated heavily in the medial part of laminae 7–10, RMg in the rostral hindbrain gave rise to fiber terminals in laminae 1–4 in addition to the ample terminals in the medial laminae 7–10. This is very similar to the findings in the rat [15]. The distribution of fibers arising from these nuclei are summarized in (Table 1).

**Table 1:** Distribution of fibers arising from raphe nuclei and adjacent reticular formation in the mouse spinal cord

Segment\nucleus	RMg	RPa/ROb	GiV	GiA
Cervical	7–10 > 5, 6	7–10 > 4–6	6–10 > 4, 5	1, 2, 4–10 > 3
Thoracic	7–10, IMM, ICl > 4, 5	7–10, IML, ICl, IMM > 5	6–10, IMM, IML, ICl > 3–5	1, 2, 4–10, IMM, IML, ICl > 3
Lumbar	1–2, 4–10 > 3	7–10 > 4–6	4–10 > 1–3	1–10
Sacral	1–2, 4–10, SDCom, SPSy, ICl, > 3	7–10, SPSy, ICl, SDCom > 4–6	4–10, ICl, SDCom, SPSy > 1–3	1–10, ICl, SDCom, SPSy

In the rat, raphespinal fibers and boutons are present in all laminae of the lumbar cord with a predominance in laminae 5–10. However, immunohistochemical staining with serotonin antibody revealed that only a small proportion of raphespinal fibers was serotonergic. This indicates that raphe nuclei are composed of heterogeneous neurons [14, 15]. Our immunofluorescence staining with serotonin antibody also showed few overlap of BDA labeled fibers and serotonergic ones. A retrograde study on the opossum showed that after injecting HRP into the lumbar cord with the lateral funiculus of the thoracic cord transected, the majority of labeled neurons were in ROb and adjacent reticular formation, whereas injections of HRP into the dorsolateral funiculus of the cervical cord resulted in more labeled neurons in RMg and the ventral part of the gigantocellular reticular nucleus. Anterograde tracer injections into different parts of the raphe nuclei confirmed that raphespinal fibers mainly travel in the lateral funiculus [19], which is similar to the serotonergic fibers in the *Xenopus laevis* [13]. Their fibers terminate in all laminae except those from RPa and ROb, which do not terminate in laminae 1–3. In addition, some fibers terminate in the parasympathetic nucleus of the sacral cord [19]. This is similar to our results in the mouse except that raphespinal fibers mainly travel in the ventral funiculus in the upper segments of the spinal cord and there is shift of these fibers to the lateral funiculus in the lower segments. In the CLARITY preparation, the densely packed fiber bundle was observed in the ventromedial part of the gray matter and it issued branches along the rostrocaudal axis. These branches further gave collaterals along their paths towards the lateral motor neuron column. This has never been shown in previous studies. The serotonergic fibers were similarly distributed as those labeled by BDA but none of them were labeled by serotonin antibody. This indicates that serotonergic and non-serotonergic fibers share similar characters in their termination pattern.

In a study in the cat, it is shown that the bistability of α-motor neurons in the acute spinal transected cat restored after intravenous 5-hydroxytryptophan injections. This indicates the termination of serotonergic fibers on motor neurons [25]. The present immunofluorescence staining and CLARITY also showed the proximity of serotoninergic fiber terminals to motor neurons. In another study in the cat, muscle injections with wheat germ agglutinin conjugated HRP (WGA-HRP) revealed that neurons projecting to the motor neurons of the spinal cord in the raphe nuclei are non-monoaminergic [26]. This confirms that raphespinal neurons are heterogeneous in its composition as demonstrated by other studies [15, 22].

### Fiber terminals arising from the reticular formation adjacent to raphe nuclei

The present study showed that reticulospinal fibers arising from the alpha or ventral part of the gigantocellular reticular nucleus travel in both the ventral and lateral funiculi with a shift of predominance from the ventral funiculus to the lateral funiculus along the axis of the spinal cord, which is similar to those fibers arising from the raphe nuclei. However, the increased number of fibers and their terminals in the medial ventral horn and the ipsilateral predominance distinct them from those arising from the raphe nuclei. This is similar to previous studies [11, 27–30]. However, the opossum has a slightly different termination pattern of the reticulospinal fibers, which are more laterally distributed in the gray matter [19]. In the rat, these reticulospinal fibers mainly travel in the lateral funiculus but they terminate similarly as those fibers in the mouse [31]. The alpha and the ventral parts of the gigantocellular reticular nucleus showed great similarity in their fiber termination. However, an apparent difference also existed, which is shown by the presence of BDA labeled fibers in the superficial layers of the dorsal horn after BDA injections into the former nucleus.

Immunohistochemical and immunofluorescence studies have shown that the reticular formation adjacent to the raphe nuclei also contain serotonergic neurons and some of them project to the spinal cord [19, 22]. Based on the chemical staining of the raphe nuclei and the reticular formation, and the fiber distribution pattern in the spinal cord, it might be possible that these nuclei are functioning as an entity, responsible for pain perception, locomotion, and modulation of the sympathetic and parasympathetic activities.

### Technical consideration

The present study did not find any overlap between Alexa fluor 488 labelled serotonergic fibers and Alexa fluor 594-BDA labeled fibers, this might be due to the following reasons. Firstly, the number of fibers labeled by BDA-avidin-HRP complex is much bigger than that of BDA-avidin-Alexa fluor 594 complex. This suggests that the efficiency of Alexa fluor 594 conjugated avidin is much lower than HRP conjugated avidin. Secondly, only about half of the serotonergic neurons in the raphe nuclei project to the spinal cord [11]. In the immunofluorescence preparation, only a small number of neurons are positive for serotonin in a single section [22]. Therefore, the number of serotonergic neurons which project to the spinal cord might be small and the majority of these neurons are in a ventral portion of the raphe nuclei. It is likely that the anterograde tracer BDA was only uptaken by non-serotonergic neurons when the injection site did not involve the ventral part of the raphe nuclei. However, the CLARITY and immunofluorescence data provide complementary information about the serotonergic fibers in the spinal cord based on the fact that all these fibers are from hindbrain raphe nuclei.

### Functional significance

Raphe nuclei have been well known for their involvement in pain perception and modulation [1, 3–5, 32]. Other studies have shown that they are involved in other physiological activities.

Hentall et al. [7] pointed out that stimulating the area 0.5 mm lateral to the midline does not change the effectiveness of 5-HT release in the dorsal horn. It is also reported that RPa and its surrounding magno and gigantocellular reticular nuclei project to the motor neurons of the deltoid muscle [26]. Some raphespinal fibers arise from large neurons, which suggests that raphespinal might be part of the reticulospinal system involved in the locomotion control. In the frog, a pharmacological study showed that the serotonergic fibers are involved in the development of locomotor circuit [33].

It is reported that stimulating RMg results in increased release of excitatory amino acids, glycine and serotonin [3]. Others reported that the serotonergic system controls the terminal excitability of a variety of afferent groups [34–36]. In combination with the raphespinal sympatho-inhibitory fiber terminals in the cervical cord [18], it is suggested that raphe nuclei are involved in diverse physiological activities not only through their direct connections with the spinal cord, but also through connections with other brain regions.

The reticulospinal neurons of the gigantocellular reticular nucleus (including GiA and GiV) play an important role in sleep wake cycle, especially in rapid eye movement (REM) phase [37]. These neurons are active during this REM phase, and their activity increased in awake animals as their motor activity increases [38–40]. This might be explained by their the axonal terminals in the motor neuron columns. Due to their termination in the medial motor neuron column, they might be important for axial motor activity control and orientation response as observed by [38–40].

## Conclusion

Serotonergic and non-serotonergic fibers arising from the hindbrain raphe and reticular nuclei had similar termination pattern in the mouse spinal cord with subtle difference.

## Methods

### Animals

All animal procedures were reviewed and approved by the Animal Care and Ethics Committee of The University of New South Wales (14/94A-Mammalian Brain Structure in Health and Disease) in accordance with the Council Directive 2010/63EU of the European Parliament and the Council of 22 September 2010 on the protection of animals used for scientific purposes. Twenty-eight C57BL/6 mice of 12–14 weeks of age, weighing 25–30 g were used. The mice were obtained from the Animal Resource Center in Western Australia.

### Anterograde tracing

Mice were anaesthetised with an intraperitoneal injection of ketamine (80 mg/kg) and xylazine (5 mg/kg) before being positioned into a mouse stereotaxic head holder (Kopf Instruments, Tujunga, CA, USA). After drilling the skull above RMg, raphe pallidus nucleus (RPa), and raphe obscurus nucleus (ROb), 20–40 nl of BDA solution (10,000 MW, Invitrogen) was injected into them with a 5 μl Hamilton syringe (15 mice). Control animals received the same tracer BDA injections either into the cisterna magna (2 mice), the adjacent medial part of the gigantocellular reticular nucleus (Gi) and the GiA/GiV (6 mice), and the cerebellum (2 mice). In each case, the syringe was left in place for 15 min after the injection. At the end of the procedure, the skin was sutured, buprenorphine was injected subcutaneously, and topical tetracycline was sprayed over the incision. Another 3 mice were used for CLARITY.

### Tissue preparation

After a survival time of 3 weeks, mice were anesthetised with a lethal dose of pentobarbital solution (0.1 ml, 200 mg/ml) and perfused through the left ventricle with 4% paraformaldehyde in 0.1 M phosphate buffer (PB). Brains and spinal cords were removed and put into 30% sucrose for 2 days before being sectioned at 40 μm using a Leica CM 1950 cryostat.

For CLARITY, mice were anaesthetised as above and perfused with the ice cold hydrogel solution by following the protocol of Chung et al. [41] (4% acrylamide, 0.05% Bis, 0.025% VA-044 initiator, 4% PFA in 1× PBS). After removing the mouse brain and spinal cord, the spinal cord was cut into 2–5 mm segments and kept in 5 ml plastic tubes at 4°C for overnight on a rotator. Subsequently, the tissue was degassed by replacing the air with nitrogen in a fume hood for 10–15 min. The tissue was then transferred to a 37°C oven for incubation on a rotator. The tissue was then removed from the gel the next day and washed with the clearing solution (0.2 M boric acid, 4% sodium dodecyl sulfate, and PH was adjusted to 8.5) until the tissue was transparent. The following protocol was based on the published one by Tomer et al. [42] and modified. After washing the lumbar cord tissue with PBST (0.1% Triton X-100) for 24 h, the tissue was incubated in the serotonin antibody solution (1:100) for 3 days on a rotator at 37°C. The tissue was subsequently washed with PBST and incubated in the Alexa fluor 594 conjugated goat anti rabbit IgG solution for 3 days before it was washed again with PBST. After 24 h, the tissue was put into 80% glycerol for homogenizing the refractive index before it was imaged with a Leica TCS SP5 multiphoton microscope using a 20× objective with NA 0.7 and a 63× objective with NA1.4 oil. Helium neon laser was used detecting the secondary labelling system. The image was scanned at 400 Hz speed at zoom 1 and dimensions of 1,024 × 1,024 (760 nm × 760 nm) with step size of 3 μm for 20× and 1 μm for 63× objective. Emitted signal was collected into HyD detectors with line accumulation of 4–8×. 3D reconstructions were processed in OsiriX software [43].

### Immunohistochemistry/immunofluorescence staining

Sections were washed in 0.1 M PB and treated with 1% H_2_O_2_ in 50% ethanol for 30 min, then they were incubated in extravidin peroxidase solution (Sigma, 1:1000) for 2 h. Sections were then visualized with 3,3′-diaminobenzidine (DAB) reaction complex (Vector lab, Burlingame, CA, USA). For immunofluorescence staining, sections were incubated in serotonin antibody (Merck Millipore, produced in rabbit, AB938, 1:2000) and subsequently in Alexa fluor 488 conjugated anti-rabbit IgG and Alexa fluor 594 conjugated extravidin (Sigma, 1:500) for 3 h. At the end of the procedure, the DAB stained sections were rinsed, mounted onto gelatinized slides, dehydrated in gradient ethanol, cleared in xylene, and coverslipped. Fluorophore labeled sections were coverslipped with a DAKO fluorescent mounting medium.

### Data analysis

DAB labeled sections were scanned with an Aperio scanner (ScanScope XT) under 20× magnification (NA = 0.75). Scanned images were opened with Imagescope software and images of different magnifications were extracted and opened with Adobe Illustrator CS6. Brain and spinal cord sections were then compared with the diagrams of the mouse brain [44] and spinal cord [45] atlases, respectively. For fluorophore labeled sections, they were imaged with a Zeiss fluorescent microscope. Two images with green and red labels of the same field were merged with Adobe Photoshop CS6.

## Additional files

Additional file 1:
**Video S1.** A general view of a lumbar segment in CLARITY. Note serotonergic fibers are found in all laminae of the gray matter but mainly in laminae 1, 2, 9, and 10. Additional file 2:
**Video S2.** A higher magnification of the dorsal horn. Note serotonergic fibers travelled along the rostrocaudal axis and terminated along their path. Additional file 3:
**Video S3.** A higher magnification of the ventral horn. Note the fiber bundle is formed in the ventromedial part of the gray matter and it gives off the first branch which extends laterally towards the lateral motor neuron column. This branch then issues smaller branches and they form an axonal tree. Up is the rostral end and right is the lateral motor neuron column.

## Abbreviations

10N: vagus nerve nucleus
BDA: biotinylated dextran amine
CLARITY: Clear, Lipid-exchanged, Acrylamide-hybridized Rigid, Imaging/immunostaining compatible, Tissue hYdrogel
DAB: diaminobenzidine
Gi: gigantocellular reticular nucleus
GiA: alpha part of the gigantocellular reticular nucleus
GiV: ventral part the gigantocellular reticular nucleus
LPGi: lateral paragigantocellular reticular nucleus
PB: phosphate buffer
REM: rapid eye movement
RMg: raphe magnus nucleus
Rob: raphe obscurus nucleus
RPa: raphe pallidus nucleus
WGA-HRP: wheat germ agglutinin conjugated HRP

## References

[1] Rivot JP, Chaouch A, Besson JM (1980). Nucleus raphe magnus modulation of response of rat dorsal horn neurons to unmyelinated fiber inputs: partial involvement of serotonergic pathways. J Neurophysiol.

[2] Zhuo M, Gebhart GF (1997). Biphasic modulation of spinal nociceptive transmission from the medullary raphe nuclei in the rat. J Neurophysiol.

[3] Sorkin LS, McAdoo DJ, Willis WD (1993). Raphe magnus stimulation-induced antinociception in the cat is associated with release of amino acids as well as serotonin in the lumbar dorsal horn. Brain Res.

[4] Siddall PJ, Polson JW, Dampney RA (1994). Descending antinociceptive pathway from the rostral ventrolateral medulla: a correlative anatomical and physiological study. Brain Res.

[5] Chebbi R, Boyer N, Monconduit L, Artola A, Luccarini P, Dallel R (2014). The nucleus raphe magnus OFF-cells are involved in diffuse noxious inhibitory controls. Exp Neurol.

[6] Rivot JP, Chiang CY, Besson JM (1982). Increase of serotonin metabolism within the dorsal horn of the spinal cord during nucleus raphe magnus stimulation, as revealed by in vivo electrochemical detection. Brain Res.

[7] Hentall ID, Pinzon A, Noga BR (2006). Spatial and temporal patterns of serotonin release in the rat’s lumbar spinal cord following electrical stimulation of the nucleus raphe magnus. Neuroscience.

[8] Bowker RM, Westlund KN, Coulter JD (1982). Origins of serotonergic projections to the lumbar spinal cord in the monkey using a combined retrograde transport and immunocytochemical technique. Brain Res Bull.

[9] Watkins LR, Griffin G, Leichnetz GR, Mayer DJ (1981). Identification and somatotopic organization of nuclei projecting via the dorsolateral funiculus in rats: a retrograde tracing study using HRP slow-release gels. Brain Res.

[10] Allen GV, Cechetto DF (1994). Serotoninergic and nonserotoninergic neurons in the medullary raphe system have axon collateral projections to autonomic and somatic cell groups in the medulla and spinal cord. J Comp Neurol.

[11] Jones SL, Light AR (1992). Serotoninergic medullary raphespinal projection to the lumbar spinal cord in the rat: a retrograde immunohistochemical study. J Comp Neurol.

[12] Wada N, Sugita S, Jouzaki A, Tokuriki M (1993). Descending projections to coccygeal spinal segments in the cat. J Anat.

[13] van Mier P, Joosten HW, van Rheden R, ten Donkelaar HJ (1986). The development of serotonergic raphespinal projections in *Xenopus laevis*. Int J Dev Neurosci.

[14] Bullitt E, Light AR (1989). Intraspinal course of descending serotoninergic pathways innervating the rodent dorsal horn and lamina X. J Comp Neurol.

[15] Jones SL, Light AR (1990). Termination patterns of serotoninergic medullary raphespinal fibers in the rat lumbar spinal cord: an anterograde immunohistochemical study. J Comp Neurol.

[16] Marlier L, Sandillon F, Poulat P, Rajaofetra N, Geffard M, Privat A (1991). Serotonergic innervation of the dorsal horn of rat spinal cord: light and electron microscopic immunocytochemical study. J Neurocytol.

[17] Morrison SF, Gebber GL (1985). Axonal branching patterns and funicular trajectories of raphespinal sympathoinhibitory neurons. J Neurophysiol.

[18] Barman SM, Gebber GL (1988). The axons of raphespinal sympathoinhibitory neurons branch in the cervical spinal cord. Brain Res.

[19] Martin GF, Cabana T, Ditirro FJ, Ho RH, Humbertson AO (1982). Raphespinal projections in the North American opossum: evidence for connectional heterogeneity. J Comp Neurol.

[20] Bowker RM, Abbott LC (1990). Quantitative re-evaluation of descending serotonergic and non-serotonergic projections from the medulla of the rodent: evidence for extensive co-existence of serotonin and peptides in the same spinally projecting neurons, but not from the nucleus raphe magnus. Brain Res.

[21] Kachidian P, Poulat P, Marlier L, Privat A (1991). Immunohistochemical evidence for the coexistence of substance P, thyrotropin-releasing hormone, GABA, methionine-enkephalin, and leucin-enkephalin in the serotonergic neurons of the caudal raphe nuclei: a dual labeling in the rat. J Neurosci Res.

[22] VanderHorst VG, Ulfhake B (2006). The organization of the brainstem and spinal cord of the mouse: relationships between monoaminergic, cholinergic, and spinal projection systems. J Chem Neuroanat.

[23] Stamp JA, Semba K (1995). Extent of colocalization of serotonin and GABA in the neurons of the rat raphe nuclei. Brain Res.

[24] Stornetta RL, Guyenet PG (1999). Distribution of glutamic acid decarboxylase mRNA-containing neurons in rat medulla projecting to thoracic spinal cord in relation to monoaminergic brainstem neurons. J Comp Neurol.

[25] Hounsgaard J, Hultborn H, Jespersen B, Kiehn O (1998). Bistability of alpha-motoneurones in the decerebrate cat and in the acute spinal cat after intravenous 5-hydroxytryptophan. J Physiol.

[26] Alstermark B, Kümmel H, Tantisira B (1987). Monosynaptic raphespinal and reticulospinal projection to forelimb motoneurones in cats. Neurosci Lett.

[27] Nyberg-Hansen R (1965). Sites and mode of termination of reticulo-spinal fibers in the cat. An experimental study with silver impregnation methods. J Comp Neurol.

[28] Jones BE, Yang TZ (1985). The efferent projections from the reticular formation and the locus coeruleus studied by anterograde and retrograde axonal transport in the rat. J Comp Neurol.

[29] Mitani A, Ito K, Mitani Y, McCarley RW (1988). Descending projections from the gigantocellular tegmental field in the cat: cells of origin and their brainstem and spinal cord trajectories. J Comp Neurol.

[30] Liang H, Watson C, Paxinos G (2015). Terminations of reticulospinal fibers originating from the gigantocellular reticular formation in the mouse spinal cord. Brain Struct Funct.

[31] Martin GF, Vertes RP, Waltzer R (1985). Spinal projections of the gigantocellular reticular formation in the rat. Evidence for projections from different areas to laminae I and II and lamina IX. Exp Brain Res.

[32] Lovick TA, West DC, Wolstencroft JH (1978). Responses of raphespinal and other bulbar raphe neurones to stimulation of the periaqueductal gray in the cat. Neurosci Lett.

[33] Sillar KT, Woolston AM, Wedderburn JF (1995). Involvement of brainstem serotonergic interneurons in the development of a vertebrate spinal locomotor circuit. Proc Biol Sci.

[34] Proudfit HK, Larson AA, Anderson EG (1980). The role of GABA and serotonin in the mediation of raphe-evoked spinal cord dorsal root potentials. Brain Res.

[35] Proudfit HK, Anderson EG (1974). New long latency bulbospinal evoked potentials blocked by serotonin antagonists. Brain Res.

[36] Martin RF, Haber LH, Willis WD (1979). Primary afferent depolarization of identified cutaneous fibers following stimulation in medial brain stem. J Neurophysiol.

[37] Steriade M, Sakai K, Jouvet M (1984). Bulbo-thalamic neurons related to thalamocortical activation processes during paradoxical sleep. Exp Brain Res.

[38] Winson J (1981). Reticular formation influence on neuronal transmission from perforant pathway through dentate gyrus. Brain Res.

[39] Quessy S, Freedman EG (2004). Electrical stimulation of rhesus monkey nucleus reticularis gigantocellularis. 1. Characteristics of evoked head movements. Exp Brain Res.

[40] Martin EM, Pavlides C, Pfaff D (2010). Multimodal sensory responses of nucleus reticularis gigantocellularis and the responses’ relation to cortical and motor activation. J Neurophysiol.

[41] Chung K, Wallace J, Kim SY, Kalyanasundaram S, Andalman AS, Davidson TJ (2013). Structural and molecular interrogation of intact biological systems. Nature.

[42] Tomer R, Ye L, Hsueh B, Deisseroth K (2014). Advanced CLARITY for rapid and high-resolution imaging of intact tissues. Nat Protoc.

[43] Rosset A, Spadola L, Ratib O (2004). OsiriX: an open-source software for navigating in multidimensional DICOM images. J Digit Imaging.

[44] Paxinos G, Franklin KBJ (2013). The mouse brain in stereotaxic coordinates.

[45] Sengul G, Watson C, Tanaka I, Paxinos G (2012). Atlas of the spinal cord of the rat, mouse, marmoset, rhesus, and human.

